# Plasma Fibrinogen Independently Predicts Hypofibrinolysis in Severe COVID-19

**DOI:** 10.3390/metabo11120826

**Published:** 2021-11-30

**Authors:** Diana Schrick, Margit Tőkés-Füzesi, Barbara Réger, Tihamér Molnár

**Affiliations:** 1 Anesthesiology and Intensive Therapy, Clinical Centre, Medical School, University of Pécs, 7624 Pecs, Hungary; schrickdiana1@gmail.com; 2 Department of Laboratory Medicine, Clinical Centre, Medical School, University of Pécs, 7624 Pecs, Hungary; tfgitta@gmail.com (M.T.-F.); reger.barbara@pte.hu (B.R.)

**Keywords:** COVID-19, platelet, IPF, hemostasis, aggregometry, viscoelastic test

## Abstract

High rates of thrombosis are present in patients with severe acute respiratory syndrome coronavirus 2 (SARS-CoV-2). Deeper insight into the prothrombotic state is essential to provide the best thromboprophylaxis care. Here, we aimed to explore associations among platelet indices, conventional hemostasis parameters, and viscoelastometry data. This pilot study included patients with severe COVID-19 (n = 21) and age-matched controls (n = 21). Each patient received 100 mg aspirin therapy at the time of blood sampling. Total platelet count, high immature platelet fraction (H-IPF), fibrinogen, D-dimer, Activated Partial Thromboplastin Time, von Willebrand factor antigen and von Willebrand factor ristocetin cofactor activity, plasminogen, and alpha2-antiplasmin were measured. To monitor the aspirin therapy, a platelet function test from hirudin anticoagulated whole blood was performed using the ASPI test by Multiplate analyser. High on-aspirin platelet reactivity (n = 8) was defined with an AUC > 40 cut-off value by ASPI tests. In addition, in vitro viscoelastometric tests were carried out using a ClotPro analyser in COVID-associated thromboembolic events (n = 8) (*p* = 0.071) nor the survival rate (*p* = 0.854) showed associations with high on-aspirin platelet reactivity status. The platelet count (*p* = 0.03), all subjects. COVID-19 patients presented with higher levels of inflammatory markers, compared with the controls, along with evidence of hypercoagulability by ClotPro. H-IPF (%) was significantly higher among non-survivors (n = 18) compared to survivors (*p* = 0.011), and a negative correlation (*p* = 0.002) was found between H-IPF and plasminogen level in the total population. The platelet count was significantly higher among patients with high on-aspirin platelet reactivity (*p* = 0.03). Neither the ECA-A10 (*p* = 0.008), and ECA-MCF (*p* = 0.016) were significantly higher, while the tPA-CFT (*p* < 0.001) was significantly lower among patients with high on-aspirin platelet reactivity. However, only fibrinogen proved to be an independent predictor of hypofibrinolysis in severe COVID-19 patients. In conclusion, a faster developing, more solid clot formation was observed in aspirin ‘non-responder’ COVID-19 patients. Therefore, an individually tailored thromboprophylaxis is needed to prevent thrombotic complications, particularly in the hypofibrinolytic cluster.

## 1. Introduction

The COVID-19 pandemic, caused by SARS-CoV-2, is a contagious potentially life-threatening disease that has caused more than five million deaths worldwide [1]. Clinical characteristics of the disease can range from mild upper tract respiratory infection to multiple organ disfunction (MODS), multiple organ failure (MOF), and fatal hypoxemic respiratory failure [2,3]. There is a great body of evidence that COVID-19 significantly affects the coagulation system, contributing to hypercoagulable states and thrombotic events. The reason for such alterations is multifactorial, including the activation of the thrombo-inflammatory cascade and endothelial dysfunction [4,5]. There is a great need to identify novel markers to stratify disease severity and predict the outcome of disease. Such attempts can not only provide deeper insight into the pathological process, but also allow more accurate triage and faster therapeutic interventions. Based on this concept, a faster correction of abnormal coagulation parameters might be associated with improved prognosis in infected patients [6,7,8]. In addition to primary hemostasis, platelets play an important role in inflammatory and immune responses. According to a recent study, platelet count per se provide valuable data in the assessment of disease severity and outcome [9]. Zhao et al. reported that an early decrease in blood platelet count was associated with poor prognosis in COVID-19 patients [10]. Hypothetically, an infection-induced cytokine storm or the virus itself directly affects bone marrow via CD13/CD66a and destroys cells and inhibits hematopoiesis [9,11,12]. In contrast, SARS-CoV-2-associated thrombocythemia has been reported too [12,13]. Platelet indices, such as immature platelet fraction (IPF, %), are valid indicators of thrombopoiesis level [14]. IPF represents young cells that have recently been released into the circulation and contain a higher concentration of ribonucleic acid than mature platelets [14]. Recently, IPF was reported as a novel early predictive marker for disease severity in patients with COVID-19 [15]. Critically ill COVID-19 patients have impaired fibrinolysis. The hypofibrinolytic state due to decreased fibrinolytic response may contribute to COVID-associated thromboembolic events. The decreased fibrinolytic response was recently defined as a lysis time (LT) >393 s. The aim of this clinical study was to explore associations among platelet indices and conventional hemorheological parameters in patients with severe SARS-CoV-2 infection and their impact on the clinical outcome [16].

The aim of this clinical study was to explore associations among platelet indices and conventional hemorheological parameters in patients with severe SARS-CoV-2 infection. In addition, the changes of platelet reactivity and fibrinolytic response contributing to the etiology of an increased thrombotic risk associated with COVID-19 were also examined here.

## 2. Results

### 2.1. Patients and Healthy Subjects

A total number of 21 COVID-19 patients (male: 12) and 21 age-matched SARS-CoV-2 PCR-negative control subjects were enrolled into this prospective observational study. All patients had SARS-CoV-2 PCR positivity, and they required intensive care with oxygen therapy with or without ventilator support. The demography, past medical history (comorbidities), and clinical data of the study population are summarized in Table 1. Patients were compared to an age-matched control group (69 years; IQR: 52–71 vs. 67 years; IQR 63–69; *p* = 0.222). The gender ratio was the same in both study groups. There was no significant difference in their body mass index (BMI). A total of 76% of enrolled patients had hypertension and 57% of them was treated for diabetes (T2DM). Not surprisingly, significantly higher erythrocyte sedimentation rates (ESR), D-dimer levels, von Willebrand factor antigen and von Willebrand factor ristocetin cofactor activities (*p* < 0.001) were observed on admission to the ICU. Furthermore, serum levels of IL-6 and ferritin also exceeded the normal laboratory range in patients, but these markers were not measured in the controls (not shown in the table).

**Figure 1:**
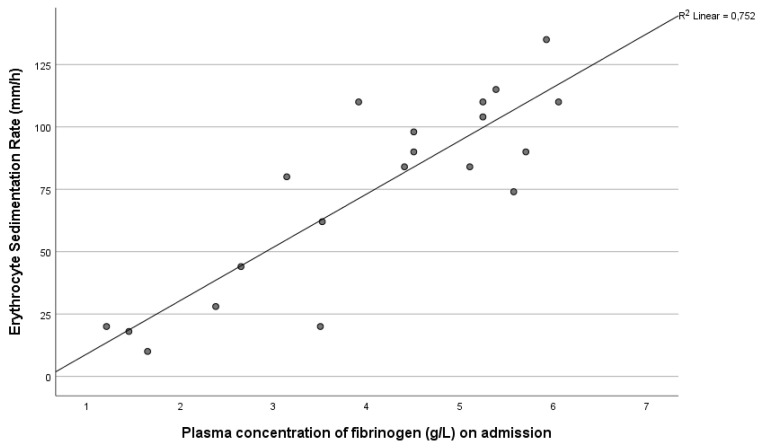
Correlation between erythrocyte sedimentation rate (ESR) and plasma fibrinogen level in COVID-19 patients on admission (*p <* 0.001).

We analyzed associations between erythrocyte sedimentation rate (ESR) and acute phase proteins, such as fibrinogen and hs-CRP. We found strong positive correlations between ESR and plasma fibrinogen levels, as well as serum hs-CRP concentration in patients, but not in healthy controls, reflecting the ongoing inflammatory response in severe SARS-CoV-2-infected patients (r = 0.812, *p* < 0.001 and r = 0.666, *p* = 0.001) (Figure 1).

A strong positive correlation was observed between von Willebrand factor antigen and plasma level of von Willebrand factor ristocetin cofactor activity in patients with severe COVID-19 (r = 0.966, *p* < 0.001). Furthermore, a positive correlation was seen between either von Willebrand factor antigen or von Willebrand factor ristocetin cofactor activity and plasma level of D-dimer (r = 0.683, *p* < 0.001; r = 0.675, *p* < 0.001, respectively—data not shown).

### 2.2. Non-Survivals vs. Survivals

**Figure 2:**
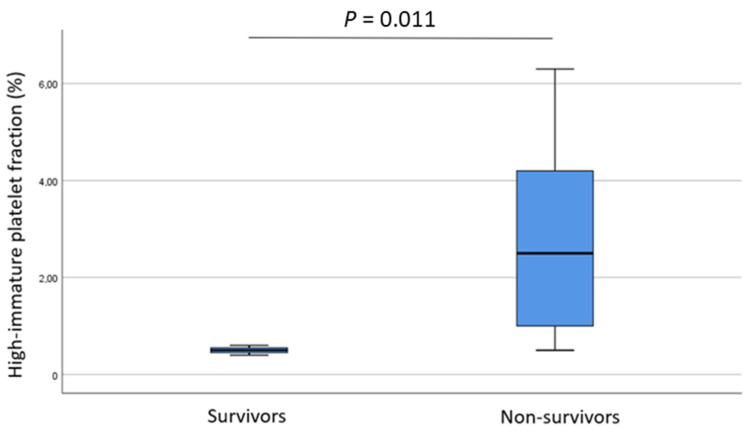
High-immature platelet fraction (%) in survivals and non-survivals.

A total of 18 patients died during intensive care, while 3 patients were discharged from hospital alive. The total platelet count showed no difference between non-survivors and survivors (*p* = 0.08). In contrast, H-IPF (%) showed significant differences when the non-survival vs. survival subgroups were compared (2.5, 1.0–4.2 vs. 0.5, 0.45–0.55; *p* = 0.011) (Figure 2). Interestingly, we detected that activated partial thromboplastin time (APTT, sec) was lower in those who died compared to survivors (13, 12.3–14.0 vs. 15.8, 14.95–26.35; *p* = 0.024).

**Figure 3:**
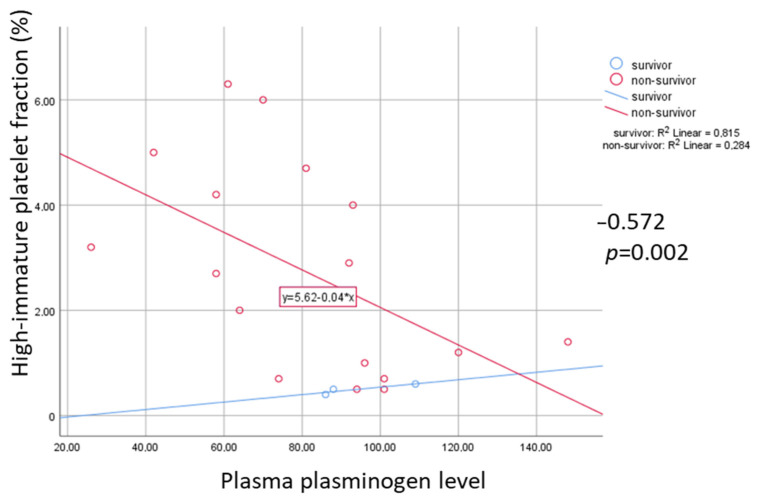
Correlation of high-immature platelet fraction (H-IPF, %) and plasma plasminogen level in survivals and non-survivals. Rho and *p* indicate negative correlation among non-survivals.

A significant negative correlation was observed between H-IPF (%) and plasma plasminogen (%) among non-survivors (r = −0.572, *p* = 0.002), but not in survivors (Figure 3).

### 2.3. ‘Responders’ vs. ‘Non-Responders’

**Figure 4:**
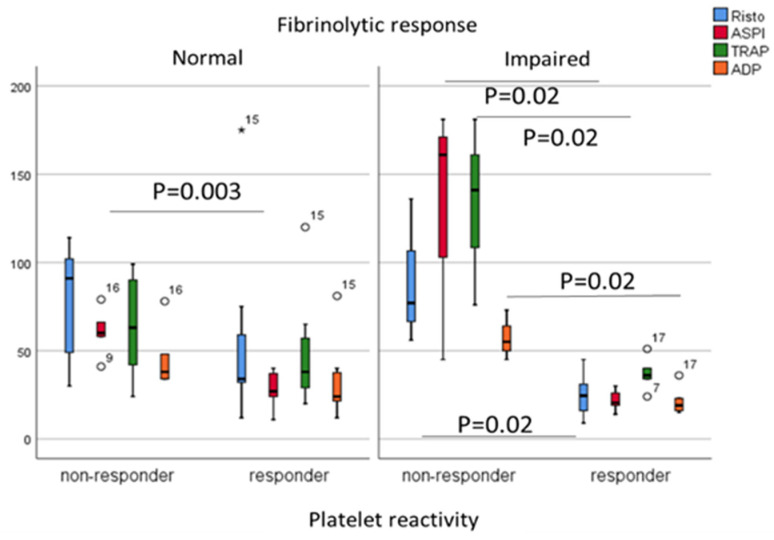
Comparisons of Risto, ASPI, TRAP and ADP tests (AUC) in patients with normal and impaired fibrinolytic response dichotomized based on their responsiveness to aspirin (responder vs. non-responder status). Mann-Whitney test (Asterix and white circles indicate extreme values).

Despite aspirin alone or in combination with enoxaparin, eight patients developed symptomatic thrombosis during their ICU stay. Patients were divided into two subgroups based on their ex vivo platelet reactivity measured by a Multiplate analyzer. High on-aspirin platelet reactivity was found in eight COVID patients using the AUC > 40 cut-off value by the ASPI test [17]. Next, COVID patients were dichotomized based on their fibrinolytic response; in patients with impaired fibrinolytic response, the AUC measured by ASPI, Risto, TRAP and ADP tests showed significant differences when aspirin responders and non-responders were compared (all *p* = 0.024, respectively) (Figure 4). Neither the thromboembolic events related to COVID-19 (*p* = 0.071), nor survival rate (*p* = 0.854) showed associations with high on-aspirin platelet reactivity status. The platelet count (*p* = 0.03), the ECA-A10 (*p* = 0.008), and ECA-MCF (*p* = 0.016) were significantly higher, while the tPA-CFT (*p* < 0.001) was significantly lower among patients with high on-aspirin platelet reactivity. In addition, the platelet count showed positive correlations with the AUC by Risto, ASPI, TRAP and ADP tests (0.500, *p* = 0.021; 0.500, *p* = 0.021; 0.760, *p* < 0.001; 0.621, *p* = 0.003, respectively), while H-IPF negatively correlated with the AUC by TRAP and ADP tests (−0.559, *p* = 0.008; −0.530, *p* = 0.013, respectively). The acute COVID-related thromboembolic events were acute coronary syndrome (n = 2), pulmonary embolism (n = 4), and ischemic stroke (n = 2). Both vWF antigen and vWF ristocetin cofactor activity showed positive correlations with H-IPF (both *p* = 0.016), and platelet count was significantly higher among patients with high on-aspirin platelet reactivity (*p* = 0.03) (data not shown).

**Figure 5:**
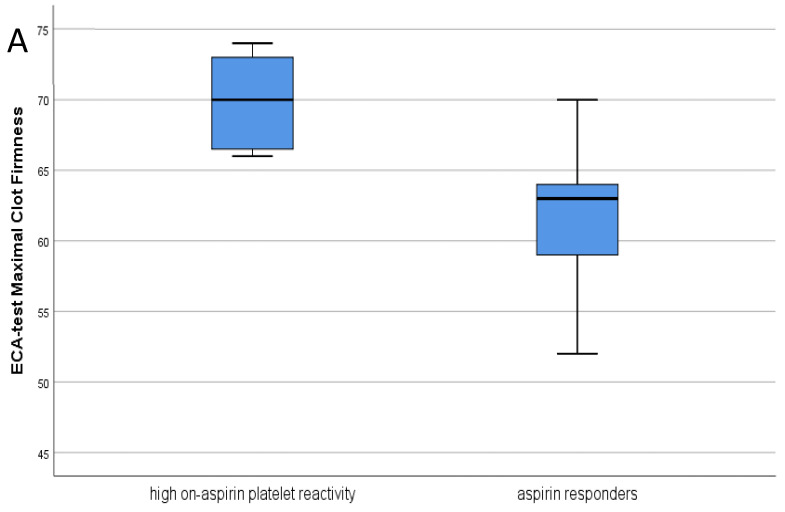

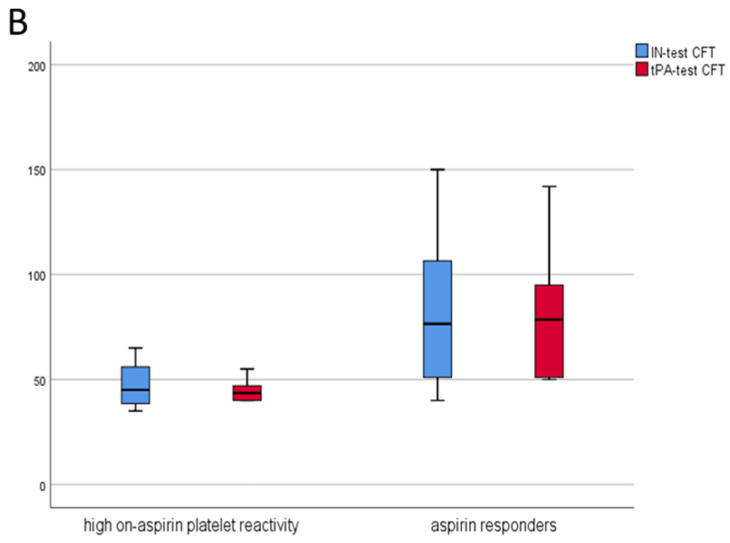
(**A**) Maximal clot firmness measured by ECA test in patients with high on-aspirin platelet reactivity vs. aspirin *‘responders’* (Mann–Whitney test, *p* = 0.016); (**B**) Clot formation time measured by IN and tPA test in patients with high on-aspirin platelet reactivity vs. aspirin *‘responders’* (Mann–Whitney test, *p* = 0.039 and *p* < 0.001, respectively).

Maximal clot firmness (MCF) was significantly higher measured by the ECA test (*p* = 0.016) in patients with high on-aspirin platelet reactivity (n = 8) compared to the ‘responder’ subgroup (n = 13), indicating larger and more solid clots despite 100 mg aspirin treatment. (Figure 5A,B). When the manufacturer’s AUC < 71 cut-off value was used in comparison, the tPA lysis time (tPA LT) tended to increase among aspirin ‘responder’ COVID-19 patients (*p* = 0.06) compared to ‘non-responders’, and eight of these patients showed features of the ‘fibrinolysis shut-down’ phenomenon.

### 2.4. Admission Platelet Count in COVID-19 Patients

**Figure 6:**
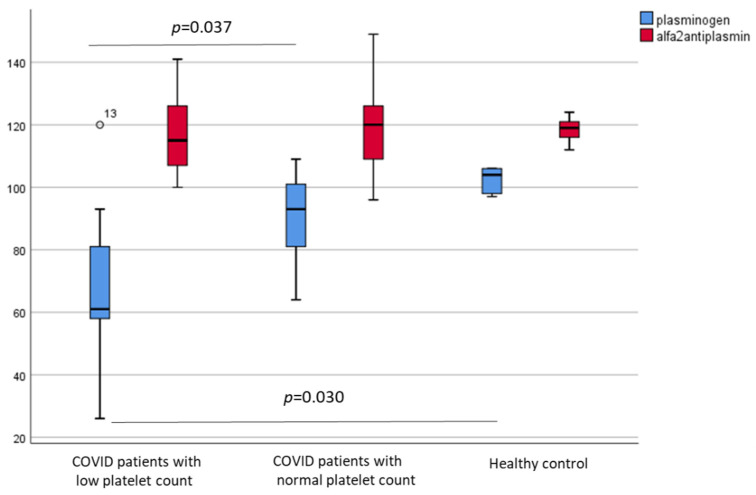
Plasminogen and alpha-2-antiplasmin levels in patients with low platelet counts (<150 g/L), with normal platelet counts (>150 g/L), and healthy controls.

**Figure 7:**
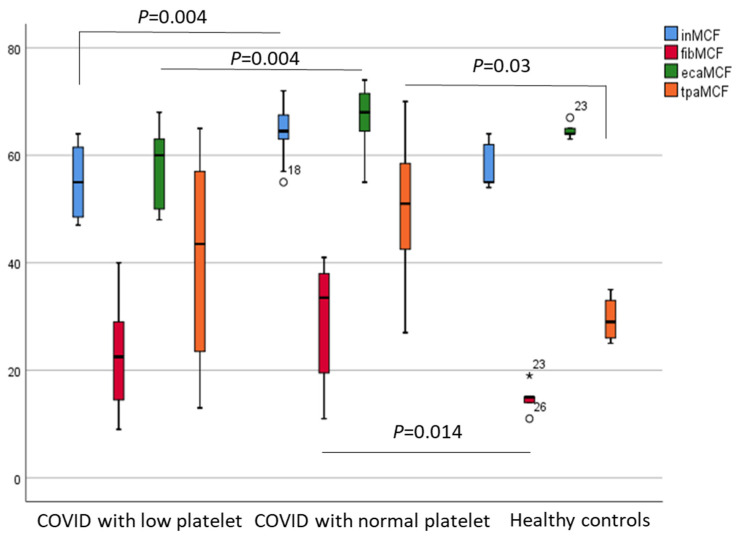
Maximal clot firmness MCF) by IN/FIB/ECA/tPA test respectively in patients with low platelet counts (<150 g/L), with normal platelet counts (>150 g/L), and healthy controls.

Next, patients were divided into two subgroups based on their platelet count on admission (thrombocytopenia <150 g/L; and normocythemia >150 g/L). Both von Willebrand factor antigen (vWF:Ag) and von Willebrand factor ristocetin cofactor activities (vWF:RCo) were significantly higher in COVID-19 patients independently from their platelet count on admission compared to healthy subjects (*p* < 0.001) (data not shown). In contrast, the plasma level of plasminogen, but not alpha-2-antiplasmin, was significantly lower among COVID-19 patients with thrombocytopenia (<150 g/L) than in patients with normal platelet counts (>150 g/L) (Figure 6). In addition, significantly lower MCF values were observed in the IN and ECA tests among patients with lower platelet counts (both *p* = 0.004), while significantly higher FIB and tPA test values were detected in patients with normal platelet counts compared to healthy controls (*p* = 0.014 and *p* = 0.03, respectively) (Figure 7).

**Figure 8:**
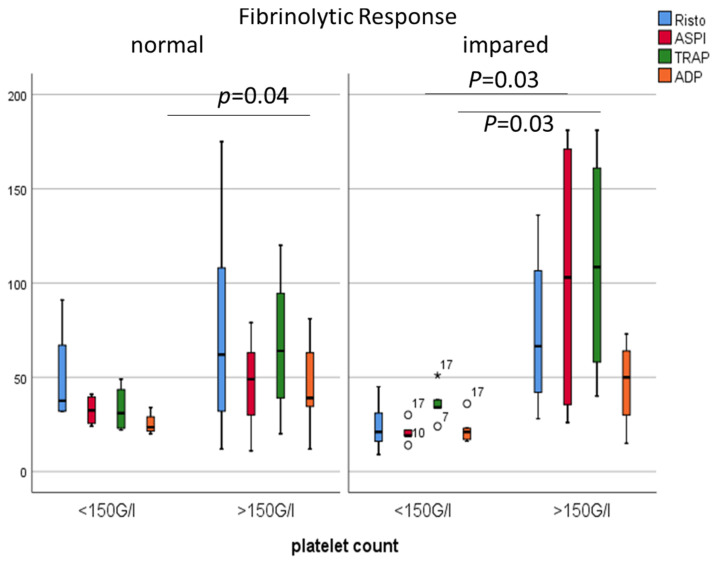
Comparisons of Risto, ASPI, TRAP and ADP tests (AUC) in patients with normal and impaired fibrinolytic response, dichotomized based on their platelet count (g/L).

Next, COVID patients were dichotomized based on their fibrinolytic response again. Thereafter, the low platelet vs. normal platelet subgroups were compared. In patients with impaired fibrinolytic response, the AUCs measured by ASPI and TRAP tests (*p* = 0.03) were significantly higher in patients with normal platelet counts. In contrast, in the normal fibrinolytic group, only the ADP test showed significant differences (*p* = 0.04) (Figure 8).

### 2.5. Independent Predictors of Ompaired Fibrinolytic Response

A separate analysis was run with hypofibrinolysis as the outcome of interest. Based on binary logistic regression analysis including age, gender, D-dimer, fibrinogen, and aspirin responsiveness based on impedance electrode aggregometry by Multiplate, only fibrinogen (OR: 3.55, 95% CI: 1.33–9.47, *p* = 0.01) proved to be an independent predictor of hypofibrinolysis. The ROC analysis of plasma fibrinogen level as a predictor of hypofibrinolysis in severe COVID patients revealed a cut-off value of 3.86 g/L (AUC of 0.800, 95% CI: 0.623–0.976; *p* = 0.006) with a 78% sensitivity and 73% specificity.

## 3. Discussion

Despite the small number of included patients, an elevated level of H-IPF (%) was found to be predictive of the fatal outcome here. Welder et al. found that elevated percentages of IPF at presentation was predictive of the length of hospitalization, the need of ICU admission, and mechanical ventilation [15]. Importantly, a higher H-IPF level was associated with lower plasminogen levels in those COVID-19 patients who died. This finding is indirectly supported by Bertolin et al., who reported increased plasminogen activator inhibitor 1 (PAI-1) activity in COVID-19 patients, that may be due to the consumption of plasminogen in association with hypercoagulability [18]. Taken together, endothelial dysfunction (elevated vWF level), with the release of fibrinolysis inhibitor PAI-1, and hyperimmune response (increased ESR, CRP, ferritin, and IL-6) with younger (higher H-IPF) activated platelets seem to be significant contributors to thrombogenesis in COVID-19. Importantly, in our cohort, the aspirin non-responder patient group presented with not only higher platelet count, but also increased platelet reactivity based on either Risto, ASPI, TRAP or ADP tests in the hypofibrinolysis (LT > 393 s) group revealed by ClotPro (likewise by Bachler et al.) [16]. Bertolin et al. also observed lower platelet reactivity based on Multiplate aggregometry compared to healthy controls, despite having higher levels of D-dimer, fibrinogen, and PAI-1, and hypercoagulability by thromboelastometry [18]. In accordance, eight COVID-patients on aspirin exhibited an increased platelet reactivity via the ASPI test (referred to in this article as ‘non-responders’). Significantly higher maximal clot firmness (MCF) was observed in ECA tests; meanwhile, significantly lower IN-, and tPA-CFT were found in ‘non-responders’ compared to ‘responders’, indicating faster developing, larger and more solid clots despite aspirin treatment. A large randomized clinical trial (*RECOVERY*) found that aspirin did not improve survival for patients hospitalized with COVID-19 [19,20]. Therefore, there is an urgent need to identify aspirin low/non-responders providing a modified antiplatelet regime or alternative strategies (e.g., activated protein C, PAI-1 antagonists, and tissue plasminogen activators) to combat thrombosis in this disease.

Based on our findings, besides platelet count itself, the MCF depends on several other factors such as plasminogen and the von Willebrand factor. Kruse et al. observed lower levels of plasminogen, suggesting that it was integrated into the clot, but unable to disintegrate it effectively, presumably by the inhibitory effect of alpha2-antiplasmin, which makes thrombi resistant to plasmin; meanwhile, plasminogen activator inhibitor (PAI-1) inhibited the activation of tissue plasminogen activator (tPA). The net effect of these may result in the ‘fibrinolysis shut-down’ phenomenon, leading to lysis-resistant microthrombi formation in different organs, particularly in the lungs [21]. Importantly, we aimed to explore predictors of impaired fibrinolytic response and found only fibrinogen with an OR: 3.55 as an independent predictor of hypofibrinolysis.

Moreover, ESR was found to be significantly higher in patients with severe SARS-CoV-2 infection and it showed strong positive correlation with fibrinogen concentration. Similar data were shown by Henry et al., who performed a meta-analysis involving 21 studies showing that inflammatory markers such as ESR, CRP, serum ferritin, IL-6, procalcitonin, and IL-2R were significantly elevated in patients with severe and fatal COVID-19 [22]. Another systematic review and meta-analysis detected that ESR positively correlated with COVID-19 severity [23]. CRP is an exquisitely sensitive systemic marker of acute-phase response in inflammation, infection, and tissue damage [24]. Elevation in serum CRP levels has been suggested in several studies as a reliable indicator of the presence and severity of SARS-CoV-2 infection [25,26,27]. D-dimer arises from the lysis of cross-linked fibrin and indicates the activation of coagulation and fibrinolysis [25,28]. Although the tPA lysis time (tPA LT) tended to be increased among aspirin ‘non-responder’ COVID-19 patients (*p* = 0.06), the D-dimer concentration was not different between ‘responder’ and ‘non-responder’ subgroups. There was no difference in D-dimer concentrations between survival and non-survival subgroups in our cohort as well (also see limitations of our study). In contrast, larger studies reported D-dimer level as a predictor for mortality. Zhang et al. stated that it is an independent factor of all-cause death in hospitalized patients with COVID-19 [29]. Regarding the kinetics, Corrado et al. found that non-survivals had rapidly increasing D-dimer levels [30]. Due to the very low survival rate of our cohort, independent predictors of mortality could not be analyzed here.

Despite the fact that we detected reduced activated partial thromboplastin time (APTT) in non-survivors, in a recent Dutch study evaluating ICU patients with COVID-19, prolongation of the prothrombin time >3 s and activated partial thromboplastin time >5 s were found to be independent predictors of thrombotic complications [31]. In our cohort, the thromboembolic complication was only associated with a reduced clotting time in the FIB test.

In fact, a high number of patients with COVID-19 die due to thromboembolic complications. Della-Morte et al. hypothesized plasminogen as the precursor for fibrinolysis [32]. Our finding was supported by them, because they found that low levels of plasminogen strongly correlated with mortality. Recently, plasminogen was suggested to play a pivotal role in controlling the complex mechanisms beyond COVID-19 complications, so it could be a useful prognostic marker and a potential therapeutic target [33].

Elevated vWF levels, as we also observed in our own cohort, imply activated or damaged endothelium [34]. It would be anticipated that damaged endothelium would result in the release of ultra-large vWF multimers capable of interacting with platelets, leading to platelet activation, microthrombi, and platelet consumption [5]. In accordance, we also found positive correlations between vWF antigen and activity and H-IPF (%) among patients with high on-aspirin platelet reactivity. Studies have shown that patients with COVID-19 have significantly elevated levels of vWF antigen and activity, likely contributing to an increased risk of thrombosis [35].

In summary, a faster-developing, larger and more solid clot formation was observed in aspirin ‘non-responder’ COVID-19 patients than ‘responders’ here. Based on ClotPro analysis, the clot seemed to be resistant to lysis in the ‘non-responders’ (longer lysis), suggesting that this cluster of patients belong to the ‘hypofibrinolysis or fibrinolysis shut-down’ group, but this requires further validation. Nevertheless, several physiological aspects should also be considered in viscoelastic studies because activation of the vessel wall, the endothelium, and platelets entering the clot is not present in vitro. Nevertheless, our results suggest the necessity of an individual approach regarding antiplatelet therapy, as was recently confirmed in other vascular diseases [36,37]. Our observations deserve further validation in a larger prospective cohort, as there is an urgent need for individually tailored thromboprophylaxis to prevent fatal complications such as symptomatic thrombosis in severe COVID-19 patients.

## 4. Limitations

First, this is a small single-center study. Results need to be confirmed on a larger sample size of COVID patients with different severity clusters. Secondly, sampling at multiple time points instead of a single time could clarify whether the kinetics of such variables differ in various outcome subgroups. Thirdly, the rigid inclusion/exclusion criteria limit the generalizability of this study.

## 5. Methods

This pilot study was approved by the Hungarian Medical Research Council (20783-5/2020/EÜIG). All procedures were performed in accordance with the ethical guidelines of the 1975 Declaration of Helsinki. Written informed consent was provided by all participants or relatives before enrolment in the present study. A total of 21 patients with severe SARS-CoV-2 infection (with the inclusion criteria: requirement of O_2_ supplementation and signed informed consent) were retrospectively analyzed from a prospective database at the Coronavirus Crisis Centre of the Clinical Centre at the University of Pécs, Pécs, Hungary. Patients under 18 years old, with congenital hemostatic abnormalities, anamnestic/current malignancy, and pregnant women were excluded from the study. Patients hospitalized in the ICU were on 100 mg/d aspirin and prophylactic anticoagulation (with enoxaparin uniformly, 1x/d) based on our local therapeutic protocol. Patients who were enrolled into the study did not receive non-steroid anti-inflammatory drugs; only paracetamol was given occasionally (e.g., in case of fever) and basic analgosedation was conducted with opioids (sufentanil uniformly). Twenty-one SARS-CoV-2 PCR negative health care workers served as healthy controls. Blood samples for measurements were drawn into a closed system blood sampling tube with 3.2% Na_3_-citrate (Becton Dickinson, Diagon Ltd., Budapest, Hungary), hirudin (Sarstedt S-Monovette^®^ 1.6 mL Hirudin), and K_3_-EDTA (Becton Dickinson, Diagon Ltd., Budapest, Hungary) as anticoagulant and serum separator tubes without anticoagulant. Samples were processed within a maximum of 1 h after collection. The blood collection from volunteers was carried out through vein puncture with a 21-gauge needle into a closed system.

### 5.1. Blood Count, Platelet Count, High Immature Platelet Fraction (H-IPF) Measurement

The total blood cell count from the whole blood and the absolute neutrophil count after 1 h of sedimentation from the upper and lower part of the blood were measured on a Sysmex XN 9000 integrated automated hematology analyzer (Sysmex Co., Kobe, Japan, 2017). The platelet number (PLT-F) was measured using the fluorescent platelet channel of the analyzer. In this channel, the platelets were specifically stained intracellularly with fluorescent dye and measured on the principle of flow cytometry, analyzing the forward scattered light (FSC), side scattered light (SSC) and side fluorescent light (SFL). The platelets were counted and additionally, the plots in the area with high fluorescence intensities were separated into the immature platelet fraction and the research parameter, the high immature platelet fraction (H-IPF).

### 5.2. The Erythrocyte Sedimentation Rate (ESR)

The ESR test measures how quickly red blood cells sedimentate in the test tube. The rate at which red blood cells settle is measured as the number of millimeters of clear plasma present at the top of the column after one hour (mm/h). For the manual determination of ESR according to Westergren, we used a BD seditainer stand with an adjustable zero mark. After swiveling the tube to mix the blood sample and preparation, the tubes were immediately placed in the stand to start the measurement. After 1 h of sedimentation, the results were read.

### 5.3. Hemostasis

Fibrinogen (quantitatively determined based on the Clauss method) and Activated Partial Thromboplastin Time (APTT) were measured as part of the routine hemostasis parameters on an ACL-TOP-750 analyzer (Werfen, Hungary) with Q.F.A. Thrombin (Bovine; HemosIL^®^, Werfen, Hungary) and APTT-SP (liquid; HemosIL^®^, Werfen, Hungary) reagent, respectively.

The special hemostasis tests were measured on an ACL-TOP-500 analyzer (Werfen, Hungary). The quantitative determination of von Willebrand factor antigen (vWF:Ag) and von Willebrand factor ristocetin cofactor activity (vWF:RCo) was performed with an automated latex enhanced immunoassay, both with HemosIL^®^ reagent. For quantitative measurement of plasminogen we used an automated chromogenic assay (Plasminogen; HemosIL^®^). The quantitative determination of alpha2-antiplasmin as an important regulator of the fibrinolytic system was carried out using an automated chromogenic assay (Plasmin Inhibitor, HemosIL^®^).

To monitor the aspirin therapy, we performed a platelet function test from hirudin anticoagulated whole blood within 1 h after blood sampling on a Cobas^®^ Multiplate^®^ Analyzer (Roche Diagnostics, Mannheim, Germany) using the ASPI test (using arachidonic acid as an activator). The aggregation level was expressed as the area under the curve (AUC). The AUC was calculated by the analyzer using the product of aggregation unit (AU) × time (minutes). Given the lack of universal cut-off values, the normal aggregation range for the ASPI test was expected as AUC: 71-115U according to the manufacturer (laboratory cut off value). However, previous studies suggest that patients were considered as ‘responders’ to aspirin therapy with an AUC < 40; and ‘non-responders’ with an AUC ≥ 40. In our data set, (n = 13) were defined as ‘responders’ and (n = 8) ‘non-responders’, showing high on-aspirin platelet reactivity.

Viscoelastrometric testing was carried out on a ClotPro (DiaCare Solutions, Mumbai, India) in vitro *POCT* coagulation analyzer. It uses pipettes prefilled with starting reagents and 340 μL of citrated whole blood to initiate measurement. For measurement, it uses a stationary pin placed in a moving cup, from which the reduction in movement is detected and charted as the amplitude resulting in thrombelastometry curves. As standard tests in COVID-19 and control patients, we used the EX test (tissue factor-activated assay with polibrene), IN test (ellagic acid-activated assay), FIB test (tissue factor activated assay, without functional platelet), ECA test (ecarin-based assay), and tPA test (r-tPA within an extrinsic pathway-based assay). Of note, the EX test, tPA test, and FIB test contain polybrene to neutralize heparin. In each test, we recorded the next parameters which characterized the whole course of coagulation: clotting time (CT), clot formation time (CFT), α angle, “amplitude of the clot” at a given time x (A(x)), maximum clot firmness (MCF), maximum lysis (ML), and lysis time (LT). The critically ill COVID-19 patients were divided into two groups based on their fibrinolytic response. A decreased fibrinolytic response (n = 9) was defined as LT > 393 s [18,38].

### 5.4. Statistical Analysis

Statistical analysis of the collected data was evaluated by IBM SPSS Statistics^®^ 27.0. To analyze demographic and clinical factors, the chi-square test was used for categorical data. The Kolmogorov–Smirnov test was applied to test for normality of continuous variables distribution. Comparisons of continuous non-normally distributed data between COVID vs. control groups were carried out using the Mann–Whitney U-test, while COVID vs. controls with or without ASA subgroups were tested using a one-way ANOVA test. A Student’s *T*-test was used for the analysis of normally distributed continuous data. Continuous variables are reported as median and interquartile range or mean and standard error of mean (SEM). Correlation analysis was performed calculating Spearman’s correlation coefficient (rho). Correlations between variables were analyzed with univariate and multivariate linear regression with corresponding beta values and 95% confidence intervals. Multivariable logistic regression was used to identify factors independently associated with decreased fibrinolytic response defined as hypofibrinolysis. A *p* value <0.05 was considered statistically significant.

## Figures and Tables

**Table 1:** Demography, comorbidities, and admission laboratory parameters of the total study population.

	Patients n = 21	Controls n = 21	*p*
Age (y)	69 (52–71)	67 (63–69)	0.222
Male (n)	12 (50%)	11 (52%)	0.757
BMI	27 (26–33)	25 (24–26)	0.189
Hypertension	16 (76)		
Diabetes mellitus	12 (57)		
Thromboembolic event (stroke/TIA, DVT)	5 (24)		
Myocardial infarct	4 (19)		
Heart failure	1 (5)		
ESR (mm/h)	84 (36–107)	4 (4–10)	<0.001
Platelet (g/L)	214 (114–355)	261 (248–265)	0.950
IPF (%)	8.7 (6.1–12.5)	7.6 (6.7–8.2)	0.753
H-IPF (%)	2.2 (0.6–4.1)	0.9 (0.8–1.0)	0.308
Fibrinogen (g/L)	5.1 (3.5–5.4)	3.2 (2.8–3.2)	0.059
D-dimer (µg FEU/L)	2296 (1415–6260)	495 (363–575)	<0.001
APTT (s)	12.9 (12.4–14.0)	26.3 (24.9–27.8)	0.002
TT (s)	34.0 (31.2–36.7)	10.4 (10.2–10.8)	<0.001
vWF:Ag (%)	488 (412–605)	109 (97–109)	<0.001
vWF:RCo (%)	399 (353–568)	104 (97–109)	<0.001
Plasminogen (%)	86 (64–96)	104 (98–106)	0.028
Alpha-2-antiplasmin (%)	118 (107–126)	119 (116–121)	1.000
hs-CRP(mg/L)	90.5 (27.7–126.1)	1.2 (0.9–1.5)	<0.001

BMI: body mass index; TIA: transient ischemic attack; DVT: deep vein thrombosis; ESR: erythrocyte sedimentation rate; IPF: immature platelet fraction, H-IPF: high-immature platelet fraction, APTT: activated partial thromboplastin time, TT: thrombin time, vWF:Ag: von Willebrand factor antigen, vWF:RCo: von Willebrand factor ristocetin cofactor activity. hs-CRP: high-sensitivity C-reactive protein. Data are presented as count (%) or median (25th–75th percentiles).

## Data Availability

The data that support the findings of this study are available on request from the corresponding author, due to high amount of raw data.
